# Employee Voice in Digital Service Platform Environments: The Roles of Perceived Digital System Support and Psychological Empowerment

**DOI:** 10.3390/bs16091466

**Published:** 2026-08-24

**Authors:** YoonSeo Kim, HyoungChul Shin

**Affiliations:** 1 Department of Hotel Culinary, Gangneung Yeongdong University, Gangneung 25521, Republic of Korea; ys.sophia.kim@gmail.com; 2 Department of Foodservice and Culinary Management, Kyonggi University, Seoul 03746, Republic of Korea

**Keywords:** digital transformation, perceived digital system support, technology acceptance model, psychological empowerment, employee voice, organizational digital systems

## Abstract

Digital transformation has reshaped work processes across the tourism industry, increasing the need to understand how employees perceive organizational digital systems and how these perceptions are associated with workplace behaviors. This study examined the relationships among perceived digital system support (PDSS), psychological empowerment, and voice behavior among tourism employees from an organizational behavior perspective grounded in the Technology Acceptance Model. Data were collected through an online survey of employees working in travel agencies, airlines, hotels, and foodservice organizations in South Korea, and 314 valid responses were analyzed using structural equation modeling. The results indicated that PDSS was positively associated with psychological empowerment, and psychological empowerment was positively associated with voice behavior. By contrast, the direct relationship between PDSS and voice behavior was not statistically significant, whereas the indirect association via psychological empowerment was significant, indicating a pattern consistent with full mediation. These findings suggest that employees’ positive perceptions of organizational digital systems show an indirect association with constructive voice behavior via psychological empowerment, particularly competence, self-determination, and impact. The study extends the application of the Technology Acceptance Model from technology acceptance to employee organizational behavior in digital work environments and highlights the importance of fostering psychologically empowering workplaces during digital transformation.

## 1. Introduction

Digital transformation has fundamentally changed how organizations create value, redesign business processes, and deliver services through digital technologies (Vial, 2021). Rather than simply introducing new technologies, organizations increasingly integrate digital systems into core operational processes to improve information sharing, decision making, and service delivery, making digital technologies an important source of strategic advantage (Bharadwaj et al., 2013). These developments have been particularly evident in the tourism industry, a labor-intensive service sector encompassing airlines, hotels, travel agencies, and foodservice organizations that continues to play a significant role in the growth of the global tourism economy (World Travel & Tourism Council, 2024). The COVID-19 pandemic further accelerated digital transformation across the tourism sector, prompting organizations to redesign value chains and organizational processes through the adoption of information and communication technologies (Gössling et al., 2020; Wurm et al., 2025). As a result, these changes have become increasingly visible through the widespread adoption of online reservation systems, mobile service channels, customer relationship management (CRM) systems, and automated self-service kiosks and point-of-sale (POS) systems (Meladze & Khakhubia, 2025; Varra & Rossi, 2019). At the industry level, tourism organizations have gradually shifted from operating aviation, lodging, travel, and foodservice services independently toward digitally connected service platforms that integrate multiple customer touchpoints and organizational functions (Sabbioni et al., 2022). Consequently, digital technology infrastructure has become a key source of organizational competitiveness in tourism (Alhelal et al., 2025). More importantly, organizations increasingly rely on digital systems to create digital work environments in which employees routinely use these technologies to perform their work and deliver customer value (Arora et al., 2025; Vlahović et al., 2024; Varra & Rossi, 2019). This shift highlights the growing importance of understanding how employees perceive organizational digital systems within digitally transformed workplaces.

Despite the rapid digitalization of tourism services, existing research has primarily examined digital technologies from the customer perspective. Previous studies have focused on consumers’ technology acceptance and usage behaviors (Li et al., 2024), the adoption of service robots and their influence on customer experiences (Moliner-Tena et al., 2025), and customer responses to online reservation platforms (Vlahović et al., 2024). Although these studies have contributed substantially to understanding technology-enabled service delivery, considerably less attention has been paid to employees who routinely use organizational digital systems as part of their daily work.

As organizations have become increasingly digitalized, organizational digital systems have become integral to employees’ work rather than merely supporting transactional efficiency. Frontline employees now interact continuously with reservation systems, customer relationship management (CRM) platforms, point-of-sale (POS) systems, and increasingly intelligent digital applications to coordinate service delivery and respond to customers in real time (Muneer et al., 2024; Vlahović et al., 2024). Consequently, digital systems function not only as operational technologies but also as organizational job resources that shape employees’ work experiences and psychological states (Bakker & Demerouti, 2017). Employees who perceive these systems as useful and supportive are more likely to feel capable of performing their work effectively and exercising greater autonomy in service delivery.

Despite these organizational changes, limited research has examined how employees’ perceptions of organizational digital systems influence their psychological states and workplace behaviors. In particular, little is known about whether perceived digital system support (PDSS) enhances employees’ psychological empowerment and subsequently encourages constructive voice behavior. Because digital transformation encompasses changes not only in technology but also in organizational processes and employee roles, understanding employees’ perceptions of organizational digital systems is essential for explaining behavioral responses within digitally transformed workplaces. Addressing this gap contributes to extending research on digital transformation from customer technology adoption to employee behavior within organizational digital environments.

Despite this research gap, prior studies have largely relied on the Technology Acceptance Model (TAM) to explain individuals’ acceptance and use of digital technologies (Davis, 1989). Although the TAM has been widely adopted to explain technology acceptance across various contexts, its primary focus on individual cognitive evaluations provides only a partial understanding of how organizational digital systems influence employees’ psychological states and workplace behaviors after technology has become embedded in daily work. As digital transformation reshapes organizational processes and work environments (Vial, 2021), digital systems increasingly function not merely as technologies to be accepted but as organizational resources that support employees in performing their jobs effectively (Bharadwaj et al., 2013). These observations suggest that the explanatory scope of the TAM can be broadened beyond technology acceptance to better understand employee behavior in digitally transformed organizations.

Building on this perspective, the present study adopts the TAM as its theoretical foundation while extending its application to the context of organizational behavior. Employees initially evaluate organizational digital systems in terms of their usefulness and ease of use, and these cognitive evaluations provide the basis for how digital systems are interpreted within the workplace (Davis, 1989). When employees perceive digital systems as useful and easy to use, they are more likely to regard them as resources that facilitate job performance rather than simply technological tools (Yu & Arshad, 2025). Such positive perceptions are expected to strengthen employees’ psychological empowerment by enhancing their sense of competence, autonomy, and influence over their work (Cao et al., 2025; Spreitzer, 1995).

Based on this theoretical framework, this study focuses on psychological empowerment and voice behavior as key outcomes of employees’ perceptions of organizational digital systems. Psychological empowerment represents a multidimensional motivational state characterized by meaning, competence, self-determination, and impact (Spreitzer, 1995). Employees who experience higher levels of psychological empowerment are more likely to engage proactively in their work and contribute to organizational improvement (Muneer et al., 2024; Raub & Robert, 2013). Voice behavior refers to employees’ discretionary expression of ideas, concerns, or suggestions intended to improve organizational functioning (Van Dyne & LePine, 1998). In tourism organizations, where frontline employees continuously interact with customers while using organizational digital systems, voice behavior has become increasingly important for service improvement, organizational learning, and adaptive responses to rapidly changing digital environments (Morrison, 2011; Han, 2025; Muneer et al., 2024; Yang et al., 2024).

Against this background, this study investigates the relationships among perceived digital system support (PDSS), psychological empowerment, and voice behavior among frontline employees in South Korea’s tourism and foodservice industries. In particular, it examines the mediating role of psychological empowerment in explaining how employees’ perceptions of organizational digital systems are associated with voice behavior. By extending the Technology Acceptance Model from technology acceptance to employee-centered organizational behavior, this study aims to provide a broader understanding of employees’ behavioral responses within digitally transformed organizations.

This study contributes to the literature in three important ways. First, it extends the explanatory scope of the Technology Acceptance Model by demonstrating that employees’ perceptions of organizational digital systems are associated not only with technology acceptance but also with psychological empowerment and organizational behavior. Second, it identifies psychological empowerment as an important explanatory mechanism linking perceived digital system support to employees’ voice behavior. Third, these findings offer practical implications for tourism organizations by suggesting that successful digital transformation depends not only on implementing advanced digital systems but also on fostering organizational environments in which employees perceive those systems as supportive resources that enhance their work and encourage employee voice and continuous organizational improvement. 

## 2. Theoretical Background

### 2.1. Technology Acceptance Model

The TAM is a theory proposed by Davis (1989), explaining the psychological mechanisms through which individuals determine their intention to use new technologies. The core constructs of the TAM are perceived usefulness, defined as the extent to which using a system enhances job performance, and perceived ease of use, defined as the extent to which system use requires minimal effort (Davis, 1989; Venkatesh & Bala, 2008). This concept is based on the theory of reasoned action (Ajzen & Fishbein, 1980), which posits that external factors influence perceptions, which, in turn, lead to attitudes or behaviors (Davis et al., 1989). Thus, the characteristics of the system do not immediately lead to action, but individuals’ acceptance of it plays a crucial role (Venkatesh et al., 2003).

In the tourism and hospitality sectors, the TAM has been widely used to explain whether consumers accept technology in the context of online reservation systems, mobile services, and various digital platforms (Varra & Rossi, 2019). However, previous research has focused on whether to use or accept technology (King & He, 2006), which partially explains the psychological processes through which tourism and foodservice employees use digital systems daily within organizations, thereby influencing organizational behavior.

In organizational settings, digital technologies function not only as tools but also as work resources that support employees’ job performance. Employees’ perceptions of the usefulness and ease of use of these systems may therefore shape their psychological experiences and subsequent workplace behavior beyond their intentions to adopt the technology. Drawing on the TAM, the present study conceptualizes perceived digital system support (PDSS) through employees’ evaluations of the usefulness and ease of use of organizational digital systems. PDSS is not proposed here as a measurement construct independent of the TAM. Rather, it represents a higher-order, context-specific conceptualization of employees’ usefulness and ease-of-use evaluations of digital systems that are already embedded in their daily work. PDSS captures the extent to which employees perceive these systems as facilitating job performance after they have become embedded in everyday work. Unlike the original TAM, which primarily explains technology acceptance intentions, the present framework focuses on employees’ evaluations of digital systems already used in their work. Consistent with the TAM, these evaluations are expected to be associated with workplace behavior and may involve employees’ psychological responses (Davis et al., 1989; Venkatesh et al., 2003). The present study accordingly examines whether psychological empowerment mediates the relationship between PDSS and voice behavior.

### 2.2. Perceived Digital System Support

Perceived digital system support (PDSS) refers to employees’ perceptions of the extent to which organizational digital systems facilitate the effective performance of their work (Venkatesh & Davis, 2000). PDSS is grounded directly in the two core evaluative dimensions of the Technology Acceptance Model (TAM), perceived usefulness and perceived ease of use (Davis, 1989; Venkatesh & Davis, 2000). Accordingly, PDSS should not be interpreted as a construct that is psychometrically independent of these TAM dimensions. In the present study, the term PDSS is used as a higher-order conceptual label for employees’ overall evaluations of the usefulness and ease of use of organizational digital systems that have already become embedded in their daily work. The distinction from the traditional TAM therefore concerns the explanatory context rather than the underlying measurement dimensions. Whereas the TAM primarily explains technology acceptance and usage intentions, the present study examines how these evaluations function in an ongoing organizational work context, in which digital systems can be perceived as resources that support task performance and work effectiveness (Bagozzi, 2007; DeLone & McLean, 2003), and how such evaluations relate to employees’ psychological empowerment and voice behavior.

PDSS also differs from several established constructs that address organizational or technological support. Perceived organizational support reflects employees’ beliefs that the organization values their contributions and cares about their well-being (Eisenberger et al., 1986; Rhoades & Eisenberger, 2002). Although such support may shape employees’ attitudes toward their work, it does not specifically address support derived from organizational digital systems (Stinglhamber & Vandenberghe, 2003). Likewise, information systems research has traditionally evaluated system quality and information quality as indicators of technical performance (DeLone & McLean, 1992, 2003). These constructs provide valuable information about the characteristics of information systems but offer less insight into how employees experience those systems while performing their work. PDSS addresses this issue by emphasizing employees’ subjective evaluations of digital systems within the organizational context in which technology and work practices interact (Orlikowski, 2000).

The relevance of PDSS is particularly evident in tourism, hospitality, and foodservice organizations. Employees in these industries rely heavily on reservation systems, customer relationship management platforms, point-of-sale systems, and other digital applications while interacting with customers and coordinating service delivery. Under these conditions, digital systems are more than operational tools; they constitute resources that help employees obtain information, make decisions, and respond efficiently to customer needs. From the perspective of the Job Demands–Resources model, such resources can reduce work demands while improving employees’ effectiveness and motivation (Bakker & Demerouti, 2017). Similar observations have been reported in the foodservice industry, where Industry 4.0 technologies and digital applications have been shown to strengthen operational capabilities and support employees throughout the service process (Romanello & Veglio, 2022).

The present study argues that employees’ evaluations of organizational digital systems have implications that extend beyond technology acceptance. Consistent with the theoretical logic of the TAM, cognitive evaluations of digital systems are expected to shape subsequent psychological and behavioral responses (Davis et al., 1989; Venkatesh et al., 2003). Employees who perceive organizational digital systems as useful and easy to use are more likely to feel capable of performing their work effectively and exercising greater control over their tasks. These perceptions are expected to strengthen psychological empowerment, which, in turn, encourages employees to express constructive suggestions and ideas that contribute to organizational improvement.

Despite the rapid digitalization of tourism organizations, research examining how employees’ TAM-based evaluations of organizational digital systems operate within ongoing organizational work contexts remains limited. Most previous studies have applied the TAM to explain technology acceptance and usage intentions, whereas relatively little attention has been paid to employees who routinely use organizational digital systems as part of their work. The present study addresses this gap by applying the theoretical foundations of the TAM to an organizational behavior context. Specifically, it examines how employees’ perceptions of organizational digital systems influence voice behavior through the mediating role of psychological empowerment.

### 2.3. Psychological Empowerment

Psychological empowerment refers to an intrinsic motivational state through which employees perceive themselves as capable of influencing their work and its outcomes (Thomas & Velthouse, 1990). Unlike formal authority or delegated power, psychological empowerment reflects employees’ internal evaluations of their work roles and their confidence in performing those roles effectively (Conger & Kanungo, 1988). Spreitzer (1995) conceptualized psychological empowerment as a multidimensional construct comprising meaning, competence, self-determination, and impact. Together, these dimensions describe how employees interpret their work, evaluate their capabilities, exercise autonomy, and perceive their influence within the organization.

The present study focuses on competence, self-determination, and impact. Competence reflects employees’ confidence in their ability to perform work successfully (Bandura, 1997), self-determination represents the degree of discretion employees experience in carrying out their work (Deci & Ryan, 2013), and impact refers to employees’ perceptions that their actions can influence organizational outcomes (Ashforth, 1989). Following Kraimer et al. (1999), these three dimensions are emphasized because they are more directly associated with employees’ proactive work behaviors. By contrast, meaning primarily reflects the personal significance attached to one’s work rather than the psychological capacity to initiate discretionary actions. Because the present study seeks to explain employees’ voice behavior, competence, self-determination, and impact provide a more appropriate operationalization of psychological empowerment.

Research has consistently shown that employees’ psychological empowerment develops when organizations provide resources, information, and conditions that enable them to perform their work effectively (Bowen & Lawler, 1992; Thomas & Velthouse, 1990). Digital systems increasingly represent one such organizational resource. In tourism, hospitality, and foodservice organizations, employees rely on reservation systems, customer relationship management platforms, point-of-sale systems, and other digital technologies to access information, coordinate services, and respond to customers. When these systems are perceived as useful and easy to use, employees are more likely to feel capable of performing their work, exercising discretion, and influencing service outcomes. Previous research likewise suggests that enhanced access to information and decision-support technologies strengthens employees’ autonomy and participation in work processes (Han, 2025).

Within the theoretical framework of the Technology Acceptance Model (TAM), employees’ cognitive evaluations of workplace technologies are expected to shape subsequent psychological responses (Davis et al., 1989; Venkatesh et al., 2003). Extending this logic to an organizational context, the present study argues that employees who perceive organizational digital systems as supportive work resources are more likely to experience higher levels of psychological empowerment. These perceptions reinforce employees’ confidence in their abilities, increase their sense of autonomy, and strengthen their belief that they can make meaningful contributions to organizational performance.

Psychological empowerment has also been identified as an important mechanism linking supportive work environments to positive organizational behaviors. In hospitality and service settings, employees who experience higher levels of psychological empowerment are more willing to contribute ideas, communicate concerns, and participate in organizational improvement initiatives (Yoo, 2017). Building on this evidence, the present study proposes psychological empowerment as a potential psychological mechanism underlying the association between perceived digital system support and employees’ voice behavior. Accordingly, psychological empowerment is conceptualized as the mediating construct linking PDSS to voice behavior.

### 2.4. Voice Behavior

Voice behavior refers to employees’ voluntary expression of ideas, concerns, or suggestions intended to improve organizational functioning or challenge existing practices (Van Dyne & LePine, 1998). Unlike routine communication, voice involves the proactive communication of constructive opinions that may improve organizational effectiveness and performance (Liang et al., 2012). Because employees often identify operational problems before managers do, voice behavior has been recognized as an important driver of organizational learning, innovation, and continuous improvement (Morrison, 2011).

Voice behavior is shaped by both individual characteristics and the organizational context in which employees perform their work. Employees are more likely to speak up when they believe that their opinions are valued and when the work environment provides sufficient support for expressing ideas (LePine & Van Dyne, 1998). Previous studies have consistently identified psychological safety, self-efficacy, leadership, and team climate as important antecedents of voice behavior because these factors reduce the perceived risks associated with speaking up (Edmondson, 1999; Detert & Burris, 2007). Voice behavior therefore reflects not only employees’ personal dispositions but also their evaluations of the organizational environment.

The importance of voice behavior is particularly evident in tourism, hospitality, and foodservice organizations, where service production and consumption occur simultaneously (Parasuraman et al., 1985). Frontline employees continuously interact with customers and are often the first to recognize operational problems, service failures, and opportunities for improvement. Their willingness to communicate these observations enables organizations to resolve service issues more quickly and improve service quality (Raub & Robert, 2013; Morrison, 2011). In these industries, voice behavior represents more than an extra-role behavior; it is an important mechanism through which organizations adapt to changing customer needs and maintain service performance.

Although voice behavior benefits organizations, employees often perceive speaking up as personally risky because it may challenge existing procedures or question managerial decisions (Raub & Robert, 2013). Employees are therefore more likely to express constructive opinions when they feel confident in their abilities, experience autonomy in performing their work, and believe that their actions can influence organizational outcomes (Spreitzer, 1995; Detert & Burris, 2007). These psychological conditions increase employees’ willingness to transform work-related observations into constructive suggestions for organizational improvement.

Perceived digital system support may strengthen these conditions by providing employees with timely information, facilitating communication, and supporting work-related decision making. When organizational digital systems are perceived as useful and easy to use, employees are better equipped to identify operational issues, exchange information, and participate in problem solving (Davis et al., 1989; Venkatesh et al., 2003). However, favorable perceptions of digital systems alone may not be sufficient to explain employees’ voice behavior. Psychological empowerment may therefore represent a potential mechanism underlying the association between perceived digital system support and constructive voice behavior.

Accordingly, the present study conceptualizes voice behavior as the behavioral outcome of employees’ perceptions of organizational digital system support and their resulting psychological empowerment. Specifically, the proposed model examines whether psychological empowerment mediates the relationship between perceived digital system support and employees’ voice behavior.

### 2.5. Hypothesis Development

#### 2.5.1. Digital System Support and Psychological Empowerment

Perceived digital system support reflects employees’ evaluations of organizational digital systems as resources that facilitate work performance. When employees perceive digital systems as useful, easy to use, and supportive of their daily tasks, they are more likely to believe that they can perform their work effectively and exercise greater control over work processes. Such perceptions provide favorable conditions for developing psychological empowerment by strengthening employees’ confidence, autonomy, and perceived influence within the organization (Spreitzer, 1995).

The relationship between organizational resources and psychological empowerment has been well established in previous research. Employees who receive sufficient information, resources, and support from their organizations generally report higher levels of competence, self-determination, and impact because these resources reduce uncertainty and enable more effective job performance (Bowen & Lawler, 1992; Thomas & Velthouse, 1990). Digital systems increasingly represent an important organizational resource in tourism, hospitality, and foodservice settings by supporting communication, information access, and operational decision making (Han, 2025).

Recent studies have likewise demonstrated that technology-based resources contribute to employees’ psychological empowerment. For example, digital competencies and technology-enabled work environments have been associated with higher levels of psychological empowerment, which subsequently enhance employees’ work performance and proactive behaviors (Lyu & Luo, 2024; Pacheco & Coello-Montecel, 2023). These findings suggest that employees who perceive greater support from organizational digital systems are more likely to develop stronger psychological empowerment.

Building on the theoretical foundations presented in Section 2.1, Section 2.2 and Section 2.3, the present study proposes that employees’ positive evaluations of organizational digital systems are positively associated with psychological empowerment. Accordingly, the following hypothesis is proposed.

**H1.**  *Perceived digital system support is positively associated with psychological empowerment.*

#### 2.5.2. Psychological Empowerment and Voice Behavior

Psychological empowerment has been widely recognized as an important antecedent of employees’ voice behavior because it enhances individuals’ confidence, autonomy, and perceived influence over organizational outcomes (Spreitzer, 1995; Zhuang et al., 2025). Employees who feel psychologically empowered are more likely to express constructive ideas and concerns because they believe that their contributions are both valuable and capable of improving organizational performance.

Previous research suggests that voice behavior emerges not only from employees’ individual characteristics but also from the interaction between psychological resources and supportive organizational environments (Pacheco & Coello-Montecel, 2023). Employees who perceive themselves as competent, autonomous, and capable of influencing organizational decisions are generally more willing to communicate suggestions, identify problems, and participate in organizational improvement initiatives (Liang et al., 2012; Thomas & Velthouse, 1990).

The importance of psychological empowerment is particularly evident in tourism, hospitality, and foodservice organizations, where frontline employees are expected to respond quickly to customer needs while maintaining service quality (Parasuraman et al., 1985). Because speaking up may involve interpersonal or organizational risk, employees are more likely to engage in voice behavior when they possess sufficient confidence in their abilities and believe that their actions can positively influence organizational outcomes (Raub & Robert, 2013; Morrison, 2011). Psychological empowerment therefore provides the motivation and sense of agency necessary for employees to transform workplace observations into constructive voice.

Building on the theoretical arguments presented in Section 2.3, the present study proposes that employees with higher levels of psychological empowerment are more likely to engage in voice behavior. Accordingly, the following hypothesis is proposed.

**H2.**  *Psychological empowerment is positively associated with voice behavior.*

#### 2.5.3. Perceived Digital System Support and Voice Behavior

Perceived digital system support may be positively associated with employees’ voice behavior because supportive digital work environments facilitate information sharing, communication, and participation in organizational decision making. When employees perceive organizational digital systems as useful and supportive, they can access work-related information more efficiently, exchange ideas more easily, and communicate suggestions through multiple digital channels. Such conditions reduce barriers to speaking up and encourage employees to participate more actively in organizational improvement.

Recent studies have shown that digital technologies facilitate knowledge sharing and collaboration by improving communication quality and information accessibility within organizations (Azarmsa et al., 2026). Digital communication platforms also encourage more open and less hierarchical interactions, creating opportunities for employees to express ideas that might otherwise remain unspoken (Hart, 2025; Konhäuser et al., 2025). As communication becomes more transparent and accessible, employees may perceive fewer interpersonal barriers when providing constructive suggestions or raising work-related concerns (Zhuang et al., 2025).

Digital work environments may therefore encourage voice behavior not only by strengthening employees’ psychological empowerment but also by directly shaping communication processes. Technology-enabled workplaces provide employees with timely information, collaborative communication channels, and greater opportunities to participate in problem solving, all of which increase the likelihood of constructive voice (Lyu & Luo, 2024; Yang et al., 2024).

Building on these arguments, the present study proposes that employees who perceive greater digital system support are more likely to engage in voice behavior. Accordingly, the following hypothesis is proposed.

**H3.**  *Perceived digital system support is positively associated with voice behavior.*

#### 2.5.4. The Mediating Role of Psychological Empowerment

The preceding hypotheses suggest that perceived digital system support is associated with both psychological empowerment and voice behavior. Beyond these direct relationships, psychological empowerment may explain how employees translate positive perceptions of organizational digital systems into constructive voice behavior. Employees who perceive digital systems as supportive of their work are more likely to feel competent, autonomous, and capable of influencing organizational outcomes, and these psychological resources increase their willingness to express ideas and suggestions for organizational improvement (Spreitzer, 1995).

Previous studies have emphasized the mediating role of psychological empowerment in linking organizational resources to positive employee outcomes. Technology-enabled work environments and digital resources have been shown to enhance employees’ psychological empowerment, which subsequently promotes proactive behaviors and organizational performance (Han, 2025; Pacheco & Coello-Montecel, 2023). Similarly, Yang et al. (2024) reported that digital leadership strengthened employees’ psychological empowerment, thereby encouraging voice behavior. Collectively, these findings suggest that psychological empowerment may represent an important mechanism underlying the association between technology-related organizational resources and employees’ discretionary behaviors. Consistent with the theoretical framework developed in Section 2.1, Section 2.2, Section 2.3 and Section 2.4, the present study proposes that psychological empowerment may account for the indirect association between perceived digital system support and voice behavior. Accordingly, psychological empowerment is expected to mediate the relationship between perceived digital system support and voice behavior.

**H4.**  *Psychological empowerment mediates the relationship between perceived digital system support and voice behavior.*

**Figure 1:**
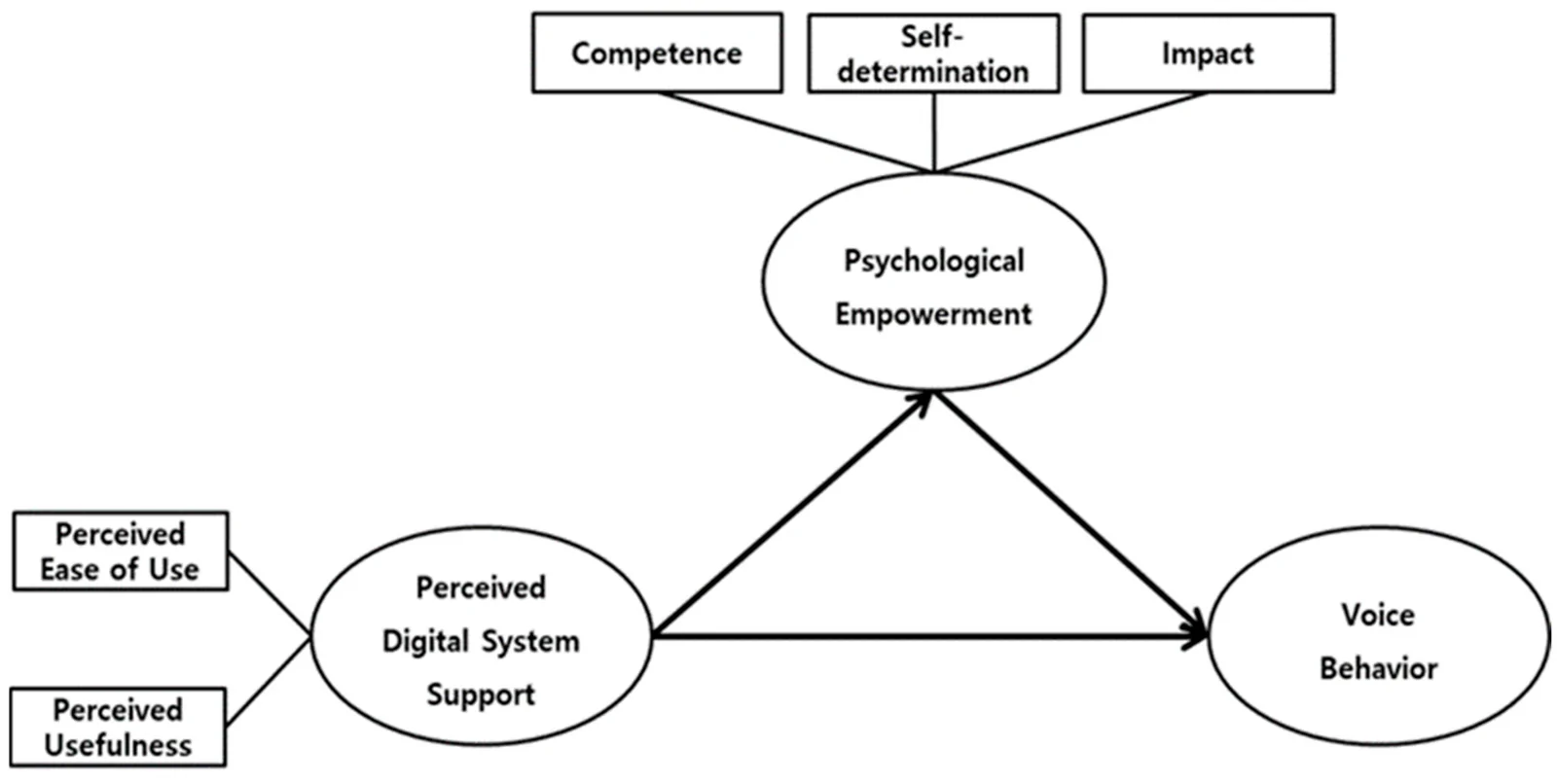
Study model.

This study aims to examine the relationships among PDSS, psychological empowerment, and voice behavior, including the proposed mediating role of psychological empowerment, among tourism employees (Figure 1).

## 3. Materials and Methods

### 3.1. Data Collection Procedure

Data were collected through an online survey of employees working in the tourism industry. Purposive sampling was employed to ensure that respondents had experience using organizational digital systems in their daily work. Eligible participants included employees from travel agencies, airlines, hotels, and foodservice organizations in South Korea who routinely used digital technologies such as reservation management systems, point-of-sale (POS) systems, kiosk devices, customer relationship management (CRM) platforms, and human resource (HR) management systems.

A screening question was included at the beginning of the questionnaire to verify respondents’ experience with organizational digital systems. Participants without such experience were automatically excluded from the survey. Data collection was conducted between 3 November and 30 November 2025, using Google Forms.

### 3.2. Participants

A total of 319 responses were obtained, of which five were removed because of incomplete or careless responses, resulting in a final sample of 314 participants. Of the final sample, 133 (42.4%) were male and 181 (57.6%) were female. The largest age group consisted of participants in their 30s (*n* = 121, 38.5%), and 224 participants (71.3%) were permanent employees. 

### 3.3. Data Analysis Procedure

To reduce the potential for common method bias, respondents were assured of anonymity and confidentiality, questionnaire items were presented in a randomized order where appropriate, and participants were informed that there were no right or wrong answers.

The data were analyzed using SPSS 30.0 and AMOS 30.0. Frequency analysis was first performed to describe respondents’ demographic characteristics. Confirmatory factor analysis was then conducted to examine the reliability and validity of the measurement model, including composite reliability, average variance extracted (AVE), and discriminant validity. Finally, structural equation modeling was employed to test the proposed hypotheses, and bootstrapping was used to assess the statistical significance of the indirect effect.

### 3.4. Instruments

PDSS refers to employees’ perceptions of the extent to which organizational digital systems (e.g., reservation management platforms, CRM tools, and HR management systems) support their work by being easy to use and helpful for improving job efficiency and performance. This perspective is based on the assumption that employees’ evaluations of digital systems, rather than the objective characteristics of the systems themselves, are more directly related to workplace attitudes and behaviors. Although operational contexts differ across tourism sectors, employees in travel agencies, airlines, hotels, and foodservice organizations commonly perform customer-contact work that requires continuous interaction with organizational digital systems. In the present study, PDSS was conceptualized from the perspective of employees’ perceptions of digital system support rather than perceived organizational support. In this study, PDSS was operationalized as a higher-order factor represented by two established TAM dimensions: perceived usefulness and perceived ease of use (Davis, 1989). This operationalization was not intended to introduce a measurement scale independent of the TAM. Rather, the two dimensions were used to capture employees’ overall evaluation of organizational digital systems that they already used routinely in performing their work. Thus, the measurement differs from conventional TAM applications primarily in its post-adoption organizational context and its criterion variables, rather than in the content of the underlying TAM dimensions. The scale consisted of six items, including three items measuring perceived usefulness and three measuring perceived ease of use, adapted to reflect the digital work environment of tourism employees.

Psychological empowerment was originally conceptualized as a four-dimensional construct comprising meaning, competence, self-determination, and impact (Spreitzer, 1995). The decision to exclude the meaning dimension was made a priori during the questionnaire design stage, before data collection and statistical analysis. Thus, the exclusion was theoretically determined rather than based on the empirical performance of the meaning items in the present dataset. No meaning items were administered to respondents. Although all four dimensions have been shown to contribute to positive work outcomes, the present study focused on competence, self-determination, and impact because these dimensions are more closely associated with employees’ perceived capability, autonomy, and influence over their work. These characteristics are particularly relevant to voice behavior, which requires employees to express ideas, challenge existing practices, and accept potential interpersonal risk. In contrast, the meaning dimension primarily reflects the extent to which employees perceive their work as personally meaningful and consistent with their values. Because the purpose of this study was to examine the psychological processes linking perceived digital system support to voice behavior, the measurement of psychological empowerment was limited to the three dimensions most directly related to employees’ willingness and perceived ability to engage in proactive workplace behaviors. This choice reflects the specific theoretical focus of the study and should not be interpreted as implying that the meaning dimension is unimportant.

Voice behavior is conceptualized as a constructive and prosocial behavior in which tourism employees voluntarily express opinions, ideas, and suggestions to improve the quality of services and operations and to address issues in organizations. This study presents voice behavior as an active, future-oriented behavior aimed to improve organizations’ development and performance, instead of as a passive expression that points out or criticizes issues. Accordingly, voice behavior was measured using items adapted from the promotive voice scale developed by Van Dyne and LePine (1998), which conceptualizes voice as the expression of constructive challenge intended to improve organizational functioning rather than merely criticize existing practices. The items were modified and supplemented to fit the job context of tourism and foodservice employees.

Finally, all measurement variables were adapted and supplemented to suit the digital work environments of tourism industry employees, based on scales with reliability and validity verified by previous studies. All items were rated using a five-point Likert-type scale (1 = not at all, 5 = very much). The complete measurement items are presented in Appendix A (Table A1).

## 4. Results

### 4.1. Demographic Characteristics

Table 1 presents the demographic characteristics of the participants. 

### 4.2. Reliability and Validity

#### 4.2.1. Confirmatory Factor Analysis

Confirmatory factor analysis was conducted to assess the structural validity of the measurement model; Table 2 presents the results. PDSS and psychological empowerment were each conceptualized as multidimensional compositional constructs and theoretically posited to possess a secondary factor structure. However, to ensure the stability and simplicity of the structural equation model analysis, the study applied the so-called multidimensional parceling (bundle) approach, which calculates the average value for each subdimension and uses it as a primary indicator. This approach is useful for reducing measurement errors and improving model fit by simplifying multidimensional compositional concepts into a single dimension. It is also widely used in structural equation research in the tourism and hotel industries.

The measurement model comprising three latent variables (i.e., PDSS, psychological empowerment, and voice behavior) exhibited excellent overall fit. Specifically, χ^2^ = 28.574 (*df* = 24, *p* = 0.237), CMIN/DF = 1.191, RMR = 0.010, GFI = 0.981, AGFI = 0.965, NFI = 0.986, IFI = 0.998, TLI = 0.997, CFI = 0.998, RMSEA = 0.025. These results indicate that the measurement model appropriately describes the data, with the fitness index meeting or exceeding the recommended criteria. In addition, in examining convergent validity, the standardized factor loadings of all measurement items reached 0.5 or higher, and the concept reliability was 0.7 or higher, thereby ensuring internal consistency of the constituent concept (Hair et al., 2016). In summary, the measurement model met the criteria for fit, reliability, and convergent validity; thus, it is deemed suitable for subsequent structural model analysis.

#### 4.2.2. Discriminant Validity

This study assessed discriminant validity using the Fornell–Larcker criterion (Fornell & Larcker, 1981). Discriminant validity refers to the extent to which theoretically distinct constructs are empirically distinguishable. According to the Fornell–Larcker criterion, discriminant validity is supported when the square root of the average variance extracted (AVE) for each construct exceeds its correlations with other constructs. As shown in Table 3, the square roots of the AVE were 0.885 for perceived digital system support, 0.884 for psychological empowerment, and 0.808 for voice behavior. The highest inter-construct correlation was 0.763 between perceived digital system support and psychological empowerment. Because the square root of the AVE for each construct exceeded its correlations with the other constructs, the measurement model satisfied the Fornell–Larcker criterion for discriminant validity.

#### 4.2.3. Common Method Bias

Given that the study relied on self-reported data collected from a single source, the potential for common method bias (CMB) was assessed using complementary diagnostic procedures. Harman’s single-factor test indicated that the first factor accounted for 45.2% of the total variance, below the commonly referenced 50% threshold (Podsakoff & Organ, 1986). In addition, all variance inflation factor (VIF) values were below 3.3 (Kock, 2015). Although these diagnostic results do not rule out the possibility of common method bias, they provide no indication that CMB was severe enough to dominate the observed relationships. Accordingly, CMB remains a methodological limitation of the present single-source design and should be considered when interpreting the findings.

### 4.3. Hypothesis Testing

Structural equation modeling was then conducted using AMOS 30.0 to test the proposed research model and hypotheses. The results of verifying the fit of the structural model indicated that the following fit indices met or exceeded the generally recommended criteria (*χ*^2^ = 28.574 [df = 24, *p* = 0.237], CMIN/DF = 1.191, RMR = 0.010, GFI = 0.981, AGFI = 0.965, NFI = 0.986, IFI = 0.998, TLI = 0.997, CFI = 0.998, RMSEA = 0.025). Thus, the structural model adequately explains the data (Hair et al., 2016).

Table 4 summarizes the results of the structural model analysis. PDSS was significantly and positively associated with psychological empowerment (β = 0.763, *p* < 0.001), supporting H1. Psychological empowerment was also significantly and positively associated with voice behavior (β = 0.751, *p* < 0.001), supporting H2. However, the direct association between PDSS and voice behavior was not statistically significant (β = 0.104, *p* > 0.05); therefore, H3 was not supported.

The mediating role of psychological empowerment was subsequently examined using the bootstrapping procedure. The indirect association between PDSS and voice behavior via psychological empowerment was significant (β = 0.573, *p* < 0.01), supporting H4. The significant indirect association, together with the non-significant direct association, suggests a pattern consistent with full mediation.

### 4.4. Robustness Analysis

To assess the robustness of the proposed mediation structure, an alternative full-mediation model was estimated by constraining the direct path from perceived digital system support (PDSS) to voice behavior to zero. The alternative model demonstrated a good fit to the data (χ^2^ = 30.433, df = 25, *p* = 0.208, CFI = 0.997, TLI = 0.996, RMSEA = 0.026, AIC = 70.433). Compared with the hypothesized model (χ^2^ = 28.574, df = 24, *p* = 0.237, CFI = 0.998, TLI = 0.997, RMSEA = 0.025, AIC = 70.574), removal of the direct path did not result in a significant deterioration in model fit (Δχ^2^ = 1.859, Δdf = 1, *p* = 0.173). In addition, the fit indices remained virtually unchanged across the two specifications, and the AIC values were nearly identical. These results indicate that the substantive findings are robust to the alternative full-mediation specification and provide additional support for the pattern of indirect association involving psychological empowerment in the relationship between PDSS and voice behavior.

## 5. Discussion

The present study examined the relationships among perceived digital system support (PDSS), psychological empowerment, and voice behavior among tourism employees. The findings showed that PDSS was positively associated with psychological empowerment, which, in turn, was positively associated with voice behavior. By contrast, the direct association between PDSS and voice behavior was not statistically significant, whereas the indirect association via psychological empowerment was significant, indicating a pattern consistent with full mediation. These findings suggest that employees’ positive perceptions of organizational digital systems are indirectly associated with constructive voice behavior via psychological empowerment. 

These findings suggest that favorable evaluations of organizational digital systems may be relevant to employee behavior beyond technology acceptance. However, the nonsignificant direct association between PDSS and voice behavior indicates that such evaluations alone may not be sufficient to explain discretionary voice behavior. Rather, the observed pattern suggests that psychological empowerment may help explain the association between employees’ evaluations of organizational digital systems and constructive voice behavior. Given that voice behavior involves interpersonal and organizational risk, employees who feel competent, autonomous, and capable of influencing organizational outcomes may be more willing to express constructive ideas and concerns (Spreitzer, 1995; Morrison, 2011; Raub & Robert, 2013).

This interpretation was further supported by the robustness analysis, in which constraining the direct PDSS–voice behavior path to zero did not significantly worsen model fit, indicating that the observed pattern of indirect association remained robust to an alternative full-mediation specification. Employees’ perceptions of digital system support appear to be indirectly associated with voice behavior via psychological empowerment. By identifying a pattern consistent with psychological empowerment as a potential explanatory mechanism underlying this association, this study contributes to a broader understanding of employee behavior in digitally transformed tourism organizations and highlights the importance of integrating technological resources with supportive organizational practices.

### 5.1. Theoretical Implications

The findings of this study provide several theoretical implications for research on digital transformation and organizational behavior.

First, this study broadens the application of the Technology Acceptance Model (TAM) by examining employees’ perceptions of organizational digital systems within the context of workplace behavior. The TAM has traditionally been used to explain individuals’ acceptance and use of technology through perceptions of usefulness and ease of use (Davis, 1989; Davis et al., 1989; Venkatesh et al., 2003). The present findings suggest that these cognitive evaluations are also relevant to organizational behavior after digital systems have become embedded in employees’ daily work. However, the non-significant direct relationship between perceived digital system support (PDSS) and voice behavior indicates that positive evaluations of digital systems alone may not be sufficient to explain discretionary workplace behaviors. Instead, the observed pattern suggests that the association between employees’ evaluations of organizational digital systems and workplace behavior may involve employees’ psychological responses.

Second, this study extends the organizational interpretation of TAM-based technology evaluations by examining perceived usefulness and perceived ease of use as indicators of how employees experience digital systems as work-related resources after these systems have become embedded in their daily work. Previous studies have primarily examined digital systems in terms of perceived usefulness and perceived ease of use (Davis, 1989; Venkatesh & Davis, 2000) or focused on technical characteristics such as system quality (DeLone & McLean, 2003). Rather than proposing PDSS as a measurement construct independent of the TAM, the present study uses PDSS as a higher-order conceptual representation of employees’ evaluations of the usefulness and ease of use of organizational digital systems within an ongoing work context. From this perspective, digital systems are interpreted as resources that facilitate employees’ work performance rather than merely as technologies to be accepted. The theoretical contribution therefore lies in extending the explanatory focus of TAM-based evaluations from technology acceptance to the psychological and behavioral consequences of routinely used organizational digital systems. This perspective is also consistent with the Job Demands–Resources framework, which suggests that organizational resources enhance employees’ motivation and work effectiveness (Bakker & Demerouti, 2017). Accordingly, these findings contribute to the literature by positioning organizational digital systems as work resources that shape employees’ psychological experiences and subsequent organizational behavior.

Third, this study identifies psychological empowerment as an important explanatory mechanism linking organizational digital systems to employees’ voice behavior. Consistent with Spreitzer (1995), employees who perceive greater competence, self-determination, and impact are more likely to engage in proactive workplace behaviors. The significant indirect association between PDSS and voice behavior via psychological empowerment, together with the nonsignificant direct association, is consistent with a full-mediation pattern. This pattern suggests that psychological empowerment may help explain the association between employees’ evaluations of organizational digital systems and constructive voice behavior. Because voice behavior often involves interpersonal risk (Morrison, 2011; Raub & Robert, 2013), this finding highlights the potential role of psychological empowerment in explaining the association between employees’ evaluations of organizational digital systems and their willingness to express constructive ideas. In doing so, this study contributes to the voice behavior literature by identifying an indirect association between TAM-based evaluations of workplace digital systems and voice behavior via employees’ psychological empowerment.

### 5.2. Practical Implications

The findings of this study provide several practical implications for tourism organizations undergoing digital transformation.

First, these findings suggest that digital transformation should not focus solely on investments in digital technologies. Many tourism organizations have introduced digital systems to improve operational efficiency and service delivery. However, the present study indicates that technological investment alone may not be sufficient to encourage employees’ constructive voice behavior. Because the direct relationship between perceived digital system support and voice behavior was not statistically significant, organizations should recognize that digital systems may be more relevant to organizational improvement when employees also experience higher levels of psychological empowerment. This finding suggests that successful digital transformation requires equal attention to both technological and human factors.

Second, tourism organizations should complement digital transformation initiatives with management practices that enhance employees’ psychological empowerment. Previous research suggests that employees who experience greater competence, self-determination, and impact are more likely to engage in proactive workplace behaviors (Thomas & Velthouse, 1990; Spreitzer, 1995). The present findings support this perspective by showing a significant indirect association between perceived digital system support and voice behavior via psychological empowerment, consistent with the proposed mediating pattern. Accordingly, managers should provide appropriate training, increase employees’ autonomy in using organizational digital systems, and establish supportive communication environments that encourage employees to share ideas for service improvement. Such practices may strengthen employees’ willingness to participate in organizational development while maximizing the value of digital technologies.

Third, these findings have particular relevance for tourism organizations because service production and consumption occur simultaneously, making frontline employees’ decisions and actions critical to service quality (Parasuraman et al., 1985; Bitner et al., 1990). Employees who actively communicate ideas and identify service problems can contribute to continuous service improvement and organizational learning (Raub & Robert, 2013; Morrison, 2011). Therefore, organizations should develop digital transformation strategies that not only improve operational efficiency but also create work environments in which employees feel confident to express constructive suggestions. Furthermore, because the use of organizational digital systems differs across travel agencies, airlines, hotels, and foodservice organizations, digital transformation initiatives should be implemented in ways that reflect the operational characteristics and customer-contact requirements of each tourism sector.

Overall, the present findings indicate that the success of digital transformation depends not only on the implementation of advanced digital systems but also on organizations’ ability to strengthen employees’ psychological empowerment and encourage constructive voice behavior. By integrating technological investment with supportive organizational practices, tourism organizations are more likely to achieve sustainable improvements in service quality and organizational performance.

### 5.3. Limitations and Future Research

This study has several limitations that should be considered when interpreting these findings and that provide directions for future research.

First, this study employed a cross-sectional research design, which limits the ability to establish causal relationships or examine changes in employees’ perceptions over time. In addition, all variables were measured using self-reported responses from a single source, which introduces the possibility of common method bias. Although Harman’s single-factor test and VIF-based diagnostics did not indicate severe common method bias, these procedures cannot rule out method-related variance. Future studies should employ longitudinal designs or multi-source data, such as supervisor-rated voice behavior, to reduce potential method bias and strengthen the robustness of the findings (Hair et al., 2016).

Second, these findings were based on employees working in tourism-related service organizations in South Korea. Although the sample included employees from travel agencies, airlines, hotels, and foodservice organizations, the data were analyzed as a single tourism sample because sector-specific comparisons were beyond the scope of the present study. Consequently, these findings should be generalized cautiously to other industries, countries, and tourism sectors. Although these tourism sectors share common characteristics, such as intensive customer contact and routine reliance on organizational digital systems, they also differ in operational processes and organizational environments. Future research should compare the proposed relationships across tourism subsectors using multi-group structural equation modeling or other comparative approaches to determine whether the findings differ across tourism contexts.

Third, employee tenure was not included as a control variable in the structural model. Although tenure was collected as an ordinal demographic characteristic, it was not incorporated into the model because the primary objective of this study was to examine the proposed theoretical relationships rather than the effects of demographic covariates. Nevertheless, employees with different levels of organizational tenure may differ in their familiarity with digital systems, psychological empowerment, and willingness to engage in voice behavior. Future research should measure tenure more precisely (e.g., years of service) and examine its role as a control variable or potential moderator in the relationships examined in this study.

Fourth, this study examined psychological empowerment as the sole mediating mechanism linking perceived digital system support and voice behavior. Although psychological empowerment provided a meaningful explanation for this relationship, other organizational and psychological mechanisms may also influence employees’ responses to organizational digital systems. Future research should extend the present model by examining both additional mediating mechanisms and relevant boundary conditions. In particular, moderators such as psychological safety, inclusive leadership, digital literacy, organizational commitment, and tourism sector characteristics may influence the strength of the proposed relationships and provide a more comprehensive understanding of employees’ voice behavior.

Finally, perceived digital system support was operationalized using perceived usefulness and perceived ease of use as its two first-order dimensions. While this specification is consistent with the theoretical foundations of the Technology Acceptance Model, relying on only two dimensions may limit the statistical stability and conceptual breadth of the higher-order construct. Digital system support in organizational settings may involve additional attributes, including system accessibility, technical support, information quality, integration across work processes, and organizational assistance in system use. Therefore, the present measure should be regarded as a parsimonious representation of employees’ perceptions of digital system support rather than an exhaustive assessment of the construct. Because PDSS was operationalized using the established TAM dimensions of perceived usefulness and perceived ease of use, the present study does not establish PDSS as a psychometrically distinct construct from these dimensions. Future research should examine the distinct predictive effects of perceived usefulness and perceived ease of use as separate constructs, compare their relative contributions to employee outcomes, and incorporate additional dimensions of organizational digital system support to further refine and validate the construct across different service and organizational contexts. 

## 6. Conclusions

This study examined the relationships among perceived digital system support (PDSS), psychological empowerment, and voice behavior among employees in the tourism industry. The findings indicated that PDSS was positively associated with psychological empowerment, which, in turn, was positively associated with voice behavior. Although the direct association between PDSS and voice behavior was not statistically significant, the indirect association via psychological empowerment was significant, indicating a pattern consistent with mediation.

This study contributes to the literature by extending the application of the Technology Acceptance Model (TAM) to an organizational behavior context. Rather than focusing on employees’ intentions to adopt new technologies, the study provides evidence that employees’ evaluations of organizational digital systems remain relevant to workplace behavior after technology has been implemented. The findings also identify psychological empowerment as a potential explanatory mechanism underlying the association between employees’ evaluations of organizational digital systems and constructive voice behavior.

From a managerial perspective, these findings suggest that successful digital transformation requires more than investments in digital technologies. Tourism organizations are more likely to realize the benefits of digital systems when technological investments are accompanied by organizational practices that strengthen employees’ psychological empowerment and encourage constructive employee participation. Overall, this study provides empirical evidence that integrating technological resources with employees’ psychological capabilities is essential for promoting proactive workplace behavior and supporting sustainable digital transformation in tourism organizations.

## Figures and Tables

**Table 1:** Demographic characteristics of the participants.

Demographic Factors	Category	*n*	Percentage (%)
Gender	Male	133	42.4
	Female	181	57.6
Age	20s	102	32.6
	30s	121	38.5
	40s	62	19.7
	50 years and older	29	9.2
Level of education	High school diploma or less	47	15.0
	Associate degree	65	20.7
	Bachelor’s degree (4-year course)	185	58.9
	Graduate degree or higher	17	5.4
Working period	Less than 1 year	70	22.3
	Between 1 and 3 years	60	19.1
	Between 3 and 5 years	50	15.9
	Between 5 and 10 years	67	21.3
	More than 10 years	67	21.3
Employment type	Permanent	224	71.3
	Nonregular	90	28.7
Total		314	100

**Table 2:** Results of confirmatory factor analysis.

Factor and Variable		Standardized Loading	S.E.	C.R.	AVE	Composite Construct Reliability (CCR)	Cronbach’s α
Perceived digital system support	PEOU	0.870	–	–	0.783	0.944	0.878
	PU	0.900	0.058	17.664 ***			
Psychological empowerment	Competence	0.870	–	–	0.782	0.963	0.915
	Self-determination	0.871	0.047	20.802 ***			
	Impact	0.912	0.045	22.576 ***			
Voice behavior	VB1	0.777	–	–	0.652	0.940	0.880
	VB2	0.824	0.067	15.408 ***			
	VB3	0.853	0.067	16.034 ***			
	VB4	0.774	0.070	14.324 ***			

χ^2^ = 28.574 (*df* = 24, *p* = 0.237), CMIN/DF = 1.191, RMR = 0.010, GFI = 0.981, AGFI = 0.965, NFI = 0.986, IFI = 0.998, TLI = 0.997, CFI = 0.998, RMSEA = 0.025. *** *p* < 0.001. PEOU = perceived ease of use, PU = perceived usefulness, S.E. = standard error, C.R. = critical ratio, AVE = average variance extracted, CCR = composite construct reliability.

**Table 3:** Discriminant validity.

Factor	Perceived Digital System Support	Psychological Empowerment	Voice Behavior
Perceived digital system support	**0.885**		
Psychological empowerment	0.763 **	**0.884**	
Voice behavior	0.605 **	0.750 **	**0.808**

Note: Diagonal values (in bold) represent the square root of the average variance extracted (AVE); values below the diagonal represent correlations between constructs. ** *p* < 0.005.

**Table 4:** Results of structural equation model analysis.

		Estimate	*t*-Value	*p*-Value	Indirect Effect		Decision
	Pathway (Hypothesis)				Estimate	*p*	
H1	PDSS → PE	0.763	13.454 ***	*p* < 0.001			Supported
H2	PE → VB	0.751	9.019 ***	*p* < 0.001			Supported
H3	PDSS → VB	0.104	1.396	0.163			Not supported
H4	PDSS → PE → VB (mediating effects of PE)	0.104	1.396	0.163	0.573 **	0.004	Supported

PDSS = Perceived digital system support; PE = psychological empowerment; VB = voice behavior. ** *p* < 0.01; *** *p* < 0.001.

## Data Availability

The data presented in this study are available on reasonable request to the corresponding author for legitimate research purposes.

## Appendix A

**Table A1:** Measurement items.

Measurement Items	
Perceived Ease of Use	Learning to operate the digital system is easy for me. My interaction with the digital system is clear and understandable. I find the digital system easy to use.
Perceived Usefulness	Using the digital system improves my job performance. Using the digital system enhances my effectiveness at work. I find the digital system useful in my job.
Competence	I am confident about my ability to do my job. I am self-assured about my capabilities to perform my work activities. I have mastered the skills necessary for my job.
Self-determination	I have autonomy in determining how I do my job. I can decide on my own how to go about doing my work. I have considerable opportunity for independence in my job.
Impact	I have significant influence over what happens in my department. My impact on what happens in my department is large. I have a great deal of control over work outcomes.
Voice Behavior	I speak up with ideas for new projects or changes. I communicate opinions about work issues even when others disagree. I proactively suggest ways to improve work procedures. I raise concerns about work-related problems.
